# Preparation and characterization of superhydrophobic surfaces based on hexamethyldisilazane-modified nanoporous alumina

**DOI:** 10.1186/1556-276X-6-487

**Published:** 2011-08-09

**Authors:** Nevin Tasaltin, Deniz Sanli, Alexandr Jonáš, Alper Kiraz, Can Erkey

**Affiliations:** 1 Department of Physics, Koç University, RumelifeneriYolu, 34450 Sariyer, Istanbul, Turkey; 2 Department of Chemical and Biological Engineering, Koç University, RumelifeneriYolu, 34450 Sariyer, Istanbul, Turkey

**Keywords:** superhydrophobic surfaces, surface modification, hexamethyldisilazane, nanoporous alumina

## Abstract

Superhydrophobic nanoporous anodic aluminum oxide (alumina) surfaces were prepared using treatment with vapor-phase hexamethyldisilazane (HMDS). Nanoporous alumina substrates were first made using a two-step anodization process. Subsequently, a repeated modification procedure was employed for efficient incorporation of the terminal methyl groups of HMDS to the alumina surface. Morphology of the surfaces was characterized by scanning electron microscopy, showing hexagonally ordered circular nanopores with approximately 250 nm in diameter and 300 nm of interpore distances. Fourier transform infrared spectroscopy-attenuated total reflectance analysis showed the presence of chemically bound methyl groups on the HMDS-modified nanoporous alumina surfaces. Wetting properties of these surfaces were characterized by measurements of the water contact angle which was found to reach 153.2 ± 2°. The contact angle values on HMDS-modified nanoporous alumina surfaces were found to be significantly larger than the average water contact angle of 82.9 ± 3° on smooth thin film alumina surfaces that underwent the same HMDS modification steps. The difference between the two cases was explained by the Cassie-Baxter theory of rough surface wetting.

## Introduction

Phenomenon of superhydrophobicity refers to the existence of very high water contact angles on solid surfaces (contact angle > 150°). This effect, which was originally observed in nature (e.g., on lotus leaves), is important for a wide range of scientific and technological applications, including development of coatings that possess self-cleaning property, reduction of viscous drag of solid surfaces subject to fluid flows, or prevention of surface fouling [1-4]. Furthermore, the ability of superhydrophobic solid surfaces with high water contact angles to support and stabilize smooth, nearly spherical aqueous droplets has led to a number of optical applications in which the surface-supported droplets act as optical resonant cavities [5]. In general, a smooth, homogeneous solid surface can be made hydrophobic by reducing its surface energy using a suitable chemical modification. However, superhydrophobic wetting regime can only be achieved by combining chemical modification of the surface with surface roughness. This idea was independently established by Wenzel [6] and Cassie and Baxter [7], and the wetting of rough surfaces has been since widely studied both theoretically and experimentally [4,8].

Recently, solids with nanometer-scale pores have become popular templates for creating superhydrophobic surfaces because of their inherent surface roughness. There exist multiple techniques for producing nanoporous surfaces such as lithography, particle deposition, template imprinting, or etching [4,8]. In this letter, we focus on nanoporous alumina-based surfaces with self-organized hexagonal pore structure prepared by electrochemical anodization of Al. With its high nanopore density, low fabrication cost, mechanical strength, and thermal stability [9], anodic alumina has been one of the most attractive nanoporous substrates used for the synthesis of superhydrophobic surfaces. In addition to its favorable material characteristics, the size and separation distance of the alumina pores can be readily adjusted by changing the electrochemical anodization conditions which allows optimizing the wetting properties of the resulting superhydrophobic surface.

Up to date various hydrophobic and superhydrophobic surfaces have been synthesized using the alumina material system. McCarley *et al*. [10] and Javaid *et al*. [11] fabricated octadecyltrichlorosilane-modified hydrophobic alumina membranes for gas-separation. Wang *et al*. [12] prepared a trichlorooctadecyl-silane-modified alumina with a water contact angle of 157°. Park *et al*. [13] fabricated heptadecafluoro-1,1,2,2-tetrahydrodecyltrichlorosilane-modified alumina membrane. Castricum *et al*. [14] modified alumina by methylchlorosilanes in toluene. Moreover, Kyotani *et al*. [15], Atwater *et al*. [16] and Yang *et al*. [17] obtained hydrophobic alumina membranes by fluorination treatment resulting in water contact angle of about 130°. Zhao *et al*. [18] and Kim *et al*. [19] fabricated a polyurethane-coated porous alumina template. The water contact angles measured in those studies were 152° and 160°, respectively. Feng *et al*. [20] modified alumina by polyethyleneimine and observed an increase of the water contact angle with the increasing immersion time in the boiling water during the surface coating procedure.

As summarized above, wetting properties of porous alumina surfaces have been modified by different chemicals including silanes. Silane molecules react strongly with the free surface hydroxyl groups of alumina, and they are among the most popular surface-modifying agents. Hexamethyldisilazane (HMDS) is a silane whose chemical activity derives from the presence of a highly reactive nitrogen atom within the compound. High silanization power of HMDS on various hydroxyl-bearing surfaces, including alumina has been demonstrated in a number of studies [21-26].

The HMDS modification of a standard alumina surface at 200°C has been investigated by Lindblad and Root [21]. They exposed the alumina samples repeatedly to the HMDS vapor and reported that new Si-OH sites are formed after each reaction treatment which acts as additional reaction sites for further silanization reactions. They also carried out experiments at different reaction temperatures and demonstrated that Si-O-Si and Al-O-Si bridges are formed via release of methyl groups with the increasing temperature [21]. Furthermore, the reaction mechanism of alumina surface with chlorotrimethylsilane was studied by Slavov *et al*. in the temperature range 80°C to 500°C. They concluded that silanization of alumina is a sequential reaction which produces methane as the only gaseous product [22]. In 1998, the same group investigated the reaction of alumina with HMDS over the temperature range 150°C to 450°C. They proposed that the initial reaction of HMDS with the alumina surface occurs by the dissociative chemisorption of HMDS via reaction of coordinatively unsaturated Al^+ ^and O-sites. Subsequent reaction of pendant -O-SiMe_3 _and -NH-SiMe_3 _groups with the surface hydroxyl groups leads to the production of ammonia, methane, hexamethyldisiloxane, and nitrogen as gaseous products [23].

In this letter, we report on the preparation and characterization of water-repellent surfaces based on HMDS vapor-treated anodic alumina with self-organized hexagonal nanopore structure. We investigate the relationship between the measured water contact angle, surface roughness, and surface chemistry, and determine the optimal silanization conditions that lead to the highest observed water contact angles. Despite the previous reports summarized above that show surface modification using HMDS, there is no account in the literature on the use of HMDS for modification of the wetting properties of nanoporous alumina surfaces. Different silanes such as chlorosilanes and fluorosilanes have been used for this purpose [10-14]. In those cases, however, hydrophobic nanoporous alumina surfaces were prepared by liquid-phase deposition in contrast to the vapor-phase deposition used in our work. Vapor-based treatment has the following important advantages over the liquid-based treatment: (1) It is simpler and shorter as it consists of fewer sample preparation stages. Prior to the liquid phase silanization, unmodified surfaces are cleaned by heating in air, boiled sequentially in hydrogen peroxide and distilled water to hydroxylate the surface, and then dried [10-14]. In contrast, our sample preparation procedure includes only boiling the sample in distilled water and drying. (2) It is performed under more controllable ambient conditions that do not require volatile organic compounds (ethanol, hexane, chloroform, toluene, etc.) for silane solutions which can affect the environment and human health. (3) It is less expensive as it requires smaller amounts of chemicals for a comparable surface coverage.

## Experimental

### Preparation of nanoporous and thin film alumina surfaces

Both nanoporous and thin film alumina surfaces were prepared through Al anodization process. Prior to anodization, high-purity Al sheets (99.999%) were annealed at 500°C for 1 h, followed by electropolishing. Alumina thin films were prepared by exposing the Al sheets to 1 wt.% phosphoric acid solution under a constant direct voltage of 194 V at 2°C for 1 hr. Nanoporous alumina samples were prepared using a two-step anodization process. First, anodic oxidation of Al was carried out as described above. Subsequently, anodically grown alumina surface layers were selectively removed by dipping the samples in the mixture of phosphoric acid (6 wt.%) and chromic acid (2 wt.%) at 50°C for 40 min. During the following second anodization, textured alumina surfaces were oxidized again at the oxidation conditions identical to the first anodization for 5 h, and thus obtained alumina was then selectively removed in 5 wt.% phosphoric acid solution at 30°C for 50 min. Scanning electron microscopy (SEM; Jeol JSM 6335, JEOL, Tokyo, Japan) was used to study the morphology of the prepared alumina nanoporous and thin film surfaces.

### Chemical modification of alumina surfaces

Surface modification of alumina by HMDS was carried out to render the prepared nanoporous and thin film alumina samples hydrophobic. In order to increase the density of the surface hydroxyl groups before the actual surface modification, the samples were first submerged in deionized H_2_O at 100°C for 1 min. Subsequently, the samples were dried at 50°C to removethe liquid water from the surfaces. The dried samples were exposed to the HMDS vapor at 100°C. The treatment was carried out in a beaker that contained liquid HMDS in equilibrium with its vapor, and the samples to be modified were placed in a sieve that was embedded at the top of the beaker. The alumina samples were exposed to the HMDS vapor for various times (4 and 9 h). This two-step surface modification procedure (exposure to boiling water followed by exposure to HMDS vapor) was applied repeatedly up to three times to both nanoporous and thin film alumina samples in order to increase the amount of hydrophobic methyl groups on the surface. Water contact angle measurements were performed on the samples after each surface modification treatment to quantify the change in hydrophobicity. Moreover, Fourier transform infrared spectroscopy-attenuated total reflectance (FTIR-ATR) analysis of the samples was performed to quantify the density of the methyl groups chemically attached to the alumina surfaces.

## Results and discussion

**Figure 1:**
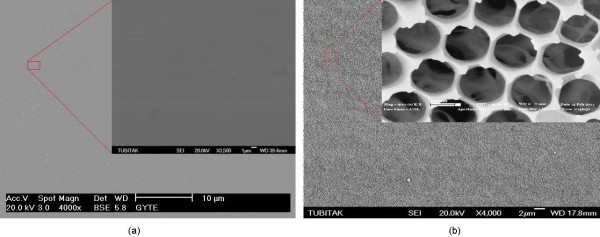
**SEM images of alumina surfaces prepared by anodic oxidation of Al**. **(a) **thin film alumina surface, **(b) **nanoporous alumina surface.

The morphology of the electrochemically prepared thin film and nanoporous alumina surfaces was characterized by SEM imaging. Figure 1 shows a typical top view of the thin film (a) and nanoporous (b) alumina surfaces. While the thin film alumina surface does not display any discernable features, hexagonal structure of circular pores that are approximately 250 nm in diameter with 300-nm interpore distances is clearly visible on the nanoporous alumina surface. The SEM image illustrates the complex 3D structure of the electrochemically prepared surface pores with pyramidal-shaped asperities protruding from the pore walls. Such complex surface topography is the key element of the resulting surface superhydrophobicity.

**Figure 2:**
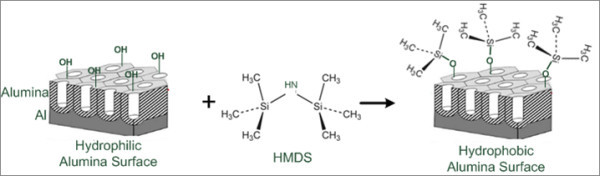
**Schematic illustration of HMDS modification process on a nanoporous alumina surface**.

To obtain hydrophobic alumina surfaces, surface modification was performed by HMDS vapor treatment with different number and duration of the treatment cycles, as described in the experimental section. During the exposure to the HMDS vapor, surface hydroxyl groups of alumina samples reacted with HMDS leading to methyl groups on the surface which bring about the hydrophobic property of the modified samples. The proposed reaction scheme for the HMDS modification process on nanoporous alumina surfaces is illustrated in Figure 2.

**Figure 3:**
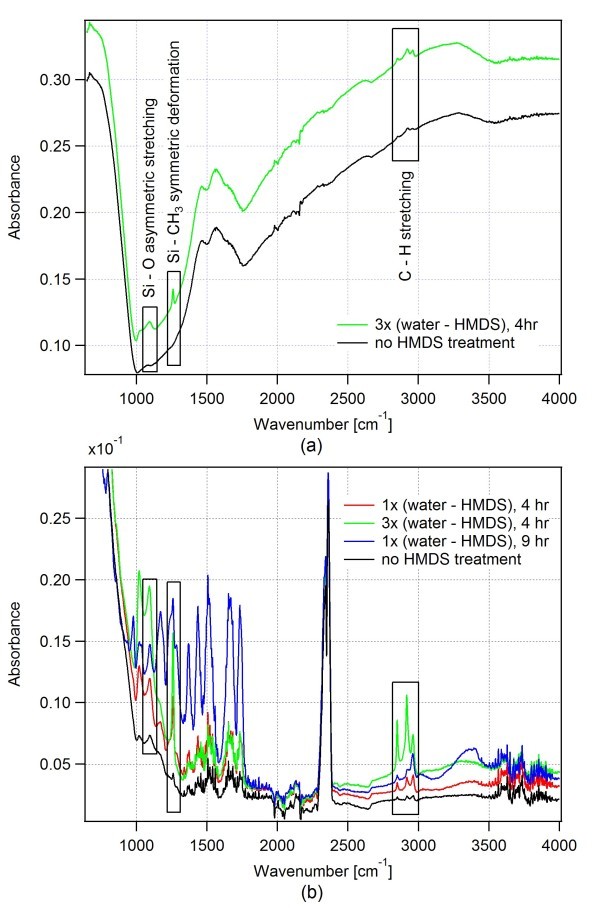
**FTIR-ATR analysis of alumina surfaces before and after HMDS modification**. **(a) **thin film alumina surface **(b) **nanoporous alumina surface.

To investigate the efficiency of the HMDS surface treatment, we performed FTIR-ATR measurements with both unmodified and modified alumina samples. Figure 3 displays the FTIR spectra obtained for the thin film (a) and nanoporous (b) alumina surfaces. The spectral peaks at 1,260 cm^-1 ^and 2,800 to 3,000 cm^-1 ^were assigned to Si-CH_3 _symmetric deformation and C-H stretching vibration, respectively. These peaks serve as markers for the presence of methyl groups on the studied surfaces. For both thin film and nanoporous alumina surfaces, the spectra of the modified samples show significant intensity of the methyl peaks which increases with prolonged HMDS treatment time. On the contrary, these peaks are virtually absent in the unmodified sample spectra. Additionally, the peak at 1,100 cm^-1 ^corresponds to the asymmetric stretching vibration of Si-O group; this spectral peak is observed at the modified samples while its amplitude at the unmodified samples is negligible. The FTIR spectra of Figure 3 indicate that HMDS reacts with the surface -OH groups of alumina samples as evident by the appearance of Si-CH_3_, C-H, and Si-O peaks at the assigned spectral positions. The incorporation of CH_3 _groups to the alumina (Al_2_O_3_) surface, thus yields the hydrophobic character of the surface.

**Figure 4:**
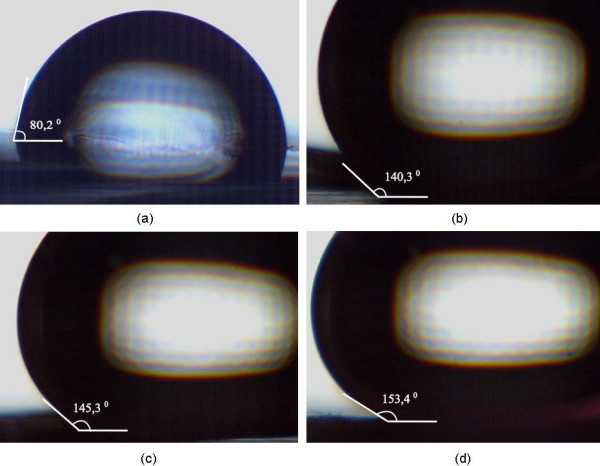
**Contact angle of water droplets on various HMDS-modified alumina surfaces**. **(a) **three times (4-h) HMDS modified thin film alumina surface, **(b) **one time (4-h) HMDS-modified nanoporous alumina surface, **(c) **three times (4-h) HMDS-modified nanoporous alumina surface, **(d) **one time (9-h) HMDS-modified nanoporous alumina surface.

The impact of the HMDS treatment conditions on the alumina surface wetting properties was characterized by the water contact angle measurements (see Figure 4). The wetting properties of unmodified thin film and nanoporous alumina surfaces were used as a reference. Both unmodified alumina surfaces were wetted completely by water and, thus, they were hydrophilic. However, after modification with HMDS, the alumina surfaces became hydrophobic due to the formation of the low energy methyl-terminated surface layer.

As clearly shown in Figure 4, the water contact angles on the HMDS-modified alumina surfaces increase with increasing HMDS treatment time. Summary of the water contact angles measured on various HMDS-modified alumina surfaces is given in Additional file 1. While the water contact angle of the HMDS-modified thin film alumina surface (three successive 4-h cycles) was only (82.9 ± 3)°, the water contact angles obtained for the nanoporous alumina samples modified in HMDS for one and three successive 4-h cycles were (139.2 ± 3)° and (145.3 ± 0.2)°, respectively. Increasing the HMDS treatment time of the nanoporous alumina surface to 9 h led to further increase of the water contact angle to (153.2 ± 2)°. These results clearly illustrate the necessity of the surface roughness in combination with a hydrophobic coating for obtaining a strongly water-repellent superhydrophobic surface.

We measured the largest water contact angle for a single stage 9-h HMDS treatment even though the total treatment time of the alumina surface is actually higher for the case of three consecutive 4-h cycles of HMDS vapor exposure. We attribute this finding to changes in the surface morphology of alumina in between consecutive HMDS deposition cycles, especially during the substrate drying step [27-29]. Since surface morphology is the key factor for achieving superhydrophobicity, morphology changes can subsequently lead to the decrease of the contact angle. We also note that-despite clearly demonstrating modification of the alumina surface by HMDS-the results of the FTIR-ATR analysis shown in Figure 3 do not allow a direct quantitative comparison of the levels of surface hydrophobicity achieved in different treatment procedures [30,31]. Hence, it is not possible to correlate simply the intensities of the HMDS characteristic peaks in the FTIR-ATR spectra and the corresponding water contact angle measurements.

The water contact angles of nanoporous alumina surfaces can be modeled using the Cassie-Baxter theory of rough surface wetting [7,8]. Within this theory, nanoporous surface is treated as being composed of two different materials: solid alumina surface with surface fractional area *f*_S _and air pockets with surface fractional area *f*_V _= 1-*f*_S_. The resulting apparent water contact angle *θ*_C _on the nanoporous alumina surface is then given by the surface fraction-weighted average of the cosines of water contact angles on a smooth alumina surface with the same chemical properties (*θ*_S _= *θ*) and air (*θ*_V _= 180°):(1)

**Figure 5:**
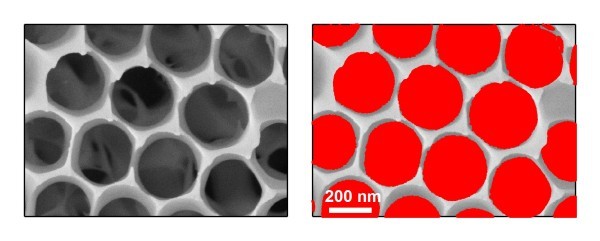
**Analysis of the solid surface fractional area of nanoporous alumina surfaces**. **(a) **High-magnification SEM image of the studied alumina surface. **(b) **Identification of the solid fraction of the surface (gray scale pixels) and air pockets (red pixels).

In order to calculate the expected value of *θ*_C _from the contact angle *θ *measured on a smooth alumina surface, solid surface fractional area *f*_S _has to be known. This can be estimated by analyzing the SEM pictures of the studied nanoporous alumina surfaces. Figure 5 shows the results of such surface fractional area analysis that provided the value of *f*_S _= 0.38. Inserting this value together with the contact angle measured on a smooth alumina surface (*θ *= 82.9° for three times water-HMDS-modified alumina thin film) into Equation 1 yields the expected *θ*_C _= 125°. In comparison, the real value of the water contact angle measured on three times water-HMDS-modified nanoporous alumina surface is *θ*_C, measured _= 145.3°.

The disagreement between the calculated and measured water contact angles stems mostly from the conservative way of estimating the solid surface fractional area: the above given value of *f*_S _corresponds to the maximal surface fraction that can be wetted and, thus, the estimated *θ*_C _represents the lower bound of the expected water contact angle. In the experiment, the true wetted fraction of the solid surface is likely smaller due to the sharp asperities protruding from the alumina surface that can serve as the real contacts supporting the droplet (see Figure 5). Such a reduction in the effective liquid-solid contact area subsequently leads to an increase of the apparent contact angle.

## Conclusion

We have described an experimental procedure for the preparation of superhydrophobic surfaces based on anodically oxidized nanoporous alumina functionalized with hexamethyldisilazane. We have characterized the water contact angles of the prepared surfaces and determined optimal experimental conditions for obtaining maximal water contact angles. Consistently with previous reports, our results have shown that both the hydrophobic surface chemistry and the nanoscale surface roughness are required for obtaining desired superhydrophobic properties. The presented procedure for the superhydrophobic surface fabrication is simple and inexpensive and, thus, it represents an interesting alternative for potential technological applications.

## Competing interests

The authors declare that they have no competing interests.

## Authors' contributions

NT carried out the preparation of the alumina surfaces and the contact angle measurements and participated in the FTIR measurements. DS carried out the HMDS modification of the alumina surfaces and participated in the analysis of the FTIR spectra. AJ participated in the FTIR measurements and the analysis of the spectra and carried out the analysis of the water contact angles on nanoporous alumina. AK and CE participated in the design of the study and coordination of the work. All authors contributed to interpretation of the results and drafting of the manuscript and they read and approved the final version.

## Supplementary Material

Additional file 1 **Water contact angles on alumina surfaces**. Contact angles of the water droplets on HMDS-modified thin film and nanoporous alumina surfaces. Click here for file
