# Supplementary figures and images for: Preparation and characterization of superhydrophobic surfaces based on hexamethyldisilazane-modified nanoporous alumina
Source: Nanoscale Res Lett. 2011 Aug 9;6(1):487. doi: 10.1186/1556-276X-6-487 (PMC3212001; doi:10.1186/1556-276X-6-487)

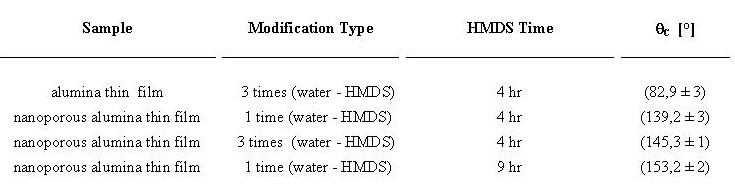

Supplement: Additional file 1 — Water contact angles on alumina surfaces. Contact angles of the water droplets on HMDS-modified thin film and nanoporous alumina surfaces. [file 1556-276X-6-487-S1.JPEG]
